# Comparison of Upper Body Joint and Hand Motions in Eating Solid Foods With Chopsticks and Semisolid Foods With a Spoon in Healthy Males and Females: Observational Study

**DOI:** 10.2196/76239

**Published:** 2026-03-06

**Authors:** Jun Nakatake, Shogo Maeda, Shigeaki Miyazaki, Hideki Arakawa, Etsuo Chosa

**Affiliations:** 1 Rehabilitation Unit, University of Miyazaki Hospital, 5200 Kihara Kiyotake-cho, Miyazaki-shi, Miyazaki, 889-1692, Japan, 81 985-85-9849, 81 985-85-9847; 2 Department of Rehabilitation Medicine, Junwakai Memorial Hospital, Miyazaki-shi, Miyazaki, Japan

**Keywords:** activities of daily living, biomechanical phenomena, neck, torso, upper extremity

## Abstract

**Background:**

Foods are not only masticated and swallowed but they also influence the choice of utensils and their use. Comparing the contexts in which different utensils are used with each food form could help in the assessment of individuals experiencing eating difficulties in the food culture unique to East Asian countries.

**Objective:**

Considering East Asian rehabilitation practices, in this study, we evaluated upper body movements involved in eating pickles (solid food) and yogurt (semisolid food) using chopsticks and a spoon, respectively.

**Methods:**

Upper body kinematics, including joint and hand spatiotemporal parameters, were quantified using a 3D inertial motion-capturing system and analyzed in healthy males (n=22; mean age 27.9, SD 5.5 years) and females (n=21; mean age 26.9, SD 4.7 years) across 4 feeding phases (reaching, picking it up, transporting, and inserting food into the mouth) by comparing utensils for eating respective food forms using the Wilcoxon signed rank test.

**Results:**

Both sexes showed smaller maximum shoulder flexion angles with chopsticks for eating solid food across all phases (Males: reaching phase, *P*<.001; picking foods up phase, *P*=.04; transport phase, *P*<.001; and mouth phase, *P*<.001. Females: reaching phase, *P*<.001; picking foods up phase, *P*=.007; transport phase, *P*<.001; and mouth phase, *P*<.001.). Elbow, forearm, and wrist ulnar deviation angle changes were smaller using chopsticks during the “picking up” phase (in both sexes, *P*<.001) compared with using a spoon. However, greater elbow joint angle changes were found during the “reaching” phase (males, *P*=.002; females, *P*=.03) and greater forearm angle changes were found during the “transporting” phase (males, *P*=.01; females, *P*=.001) with chopsticks than with spoons. Regarding hand spatiotemporal parameters, the chopstick condition involved shorter actual distances (males, *P*<.001; females, *P*<.001), lower distance efficiency (males, *P*=.04; females, *P*=.001), and slower speed (males, *P*=.001; females, *P*<.001) during food transport.

**Conclusions:**

The joint angle and hand spatiotemporal parameter characteristics observed during chopstick use for eating solid foods and spoon use for eating semisolid foods in healthy individuals could serve as reference movements in individuals with sensorimotor dysfunctions and inform the selection of adaptive utensils in rehabilitation practice.

## Introduction

In East Asian countries, such as China, Korea, and Japan, chopsticks are primarily used to take food into the mouth during meals. This utensil serves multiple functions, including picking up solid foods, cutting them into manageable pieces, and securely holding them while bringing them to the mouth. Due to their versatile functions, chopsticks have become an integral part of the food customs of these regions. To compensate for the difficulty in handling semisolid foods, a spoon is typically used. Thus, chopsticks and a spoon are commonly paired with solid and semisolid foods, respectively. Furthermore, since people in Japan have the habit of consuming soup by bringing the bowl to their mouths instead of using spoons, Japanese people more often use chopsticks than do people in other countries.

When patients undergo eating activity rehabilitation, selection of appropriate feeding utensils is essential. Patients, including those recovering from strokes, often use a spoon for its adaptability to foods that are transferred to the mouth, masticated, and then swallowed. Semisolid foods, regulated for viscosity, are commonly recommended for individuals with dysphagia [1], making the spoon a preferred adaptive device. Chopsticks are not routinely selected due to the difficulty in using them for semisolid foods. As the oral and swallowing abilities improve, patients transition from using spoons for semisolid foods to using chopsticks for solid foods. The food custom in East Asian countries is also focused on the use of chopsticks for eating solid foods, which facilitates the prospect of dining together and improving well-being. However, sensorimotor dysfunctions can impair the ability to perform feeding movements involving chopsticks and spoons. For instance, patients with right hemiparesis can use chopsticks or a spoon only with their dominant right hand if their impairment is mild [2]. Therefore, the selection of chopsticks or a spoon depends on patients’ ability to transfer, masticate, and swallow foods, as well as their limb function for manipulating utensils.

Daily movements and postures can be assessed to determine whether patients encounter difficulties performing tasks. Moreover, in the context of rehabilitation practice, assessments are needed to facilitate normal or compensatory movements or to change utensils used by patients while eating [3]. Understanding typical movements and postures when using feeding utensils aids in these assessments. Previous studies have separately reported differences in upper body joint motions across feeding phases during the normal use of chopsticks [4] and spoons [5]. A comparative study on normal upper limb kinematics has shown greater joint motion during spoon use than that during chopstick use when eating pickles, a common form of solid food [6]. However, a study investigating performance while using eating utensils reported that spoon use for noodle strips and tofu cubes prolonged eating time or led to failure to properly manipulate foods [7], suggesting that the choice of utensils should match food type.

In another aspect, proximal body movements, including the neck and trunk joint angles, tilting and rotation angles, or transfer distances, occur during eating [4,5,8-10] and tend to increase when foods are prone to be dropped [8,11]. The movements and postures of these proximal segments are important as normal functions in relation to preventing food dropping and form the basis for fine upper limb function. A recent study that simulated eating movements for fictitious foods using utensils demonstrated larger neck joint motion with a spoon than with chopsticks; however, trunk flexion angles were comparable between utensils [12]. These findings perhaps imply that using a spoon requires compensatory neck joint motion for preventing food drop and trunk stability for maintaining upper extremity functions. Using chopsticks, conversely, may not need neck compensation and may need trunk stability as when using a spoon. However, the actual movement and positioning of the upper body, including the neck and trunk, during solid food intake with chopsticks, compared with those during semisolid food intake with a spoon, remain unclear.

In addition to joint parameters, spatiotemporal parameters are valuable for assessing upper limb motions [13,14]. Considering the importance of rehabilitation practice in East Asia, understanding the normal kinematics of chopstick use while eating solid foods and spoon use while eating semisolid foods not only expands knowledge about the utensils or food forms in isolation but is also crucial, yet currently lacking. Therefore, a comparative study of these 2 food type and utensil type conditions is warranted in clinical settings, as previous research found significant head motion differences in the conditions of eating food forms with these respective utensils [8]. This previous study design is thought to be validated for assessing patients with eating difficulties such as swallowing function deficit with upper limb paresis in the recovery process.

In this study, we aimed to identify the characteristics of joint motions and spatiotemporal parameters during eating solid foods with chopsticks and semisolid foods with a spoon across feeding phases. We hypothesized that upper body joint motions for chopstick use for solid food intake could be reduced compared with those for spoon use for semisolid food intake across all feeding phases; however, the time required may be similar. We also hypothesized that the distance traveled by the hand, particularly during the reaching movements, could be greater when using chopsticks than when using a spoon. Additional parameters, such as speed, could provide further insights into kinematics during clinical observations. Consequently, in this study, we investigated the characteristics of upper body joint angles and hand spatiotemporal parameters across all feeding phases by comparing the use of chopsticks for eating solid foods and of a spoon for eating semisolid foods in healthy adults.

## Methods

### Participants

Between April 2013 and October 2017, 50 members of our institution participated per the inclusion criteria. The criteria were age 20‐39 years, right-hand dominance, and no neurological or musculoskeletal disorders. Individuals with left-hand dominance for using chopsticks or a spoon were excluded.

### Eating Items and Tasks

**Figure 1.**
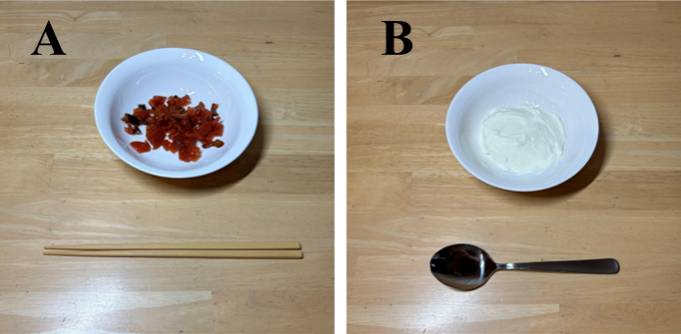
Eating items for 2 conditions. (A) Chopstick condition: small Japanese pickled vegetables in a bowl and wooden chopsticks. (B) Spoon condition: yogurt in the bowl and a stainless steel spoon.

Eating solid foods with chopsticks was performed using wooden chopsticks (22.5 centimeters in length, 8 grams in weight) and small Japanese pickled vegetables (Figure 1A). Eating semisolid foods with a spoon was performed using a stainless steel spoon (17.5 centimeters in length, 41 grams in weight) and yogurt (Figure 1B).

**Figure 2.**
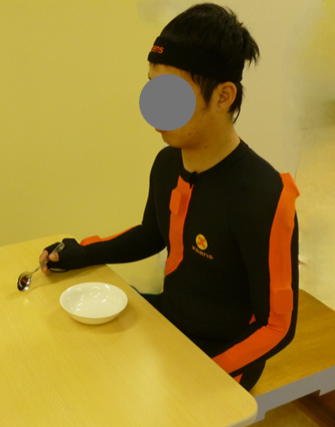
Initial position for measurements during eating tasks using utensils with the dominant right hand while the left hand rests on the left thigh. Illustrated is an example of the task of eating pickles with chopsticks or yogurt with a spoon. The participant is shown wearing a suit, grooves, and a headband, all containing inertial sensors.

The participants sat on a stool 40 centimeters in height, facing a table adjusted to their elbow height. The food bowl was placed in front of them at the same distance of the tip of the extended finger when their shoulder joint was in a neutral position and their elbow flexed at 90°. Neck and trunk movements were not constrained. The participants were asked to eat the foods using utensils held in their right hand while their left hand rested on their left thigh. They were instructed to eat at a comfortable pace and perform the movements sequentially 3 times. This frequency was to prevent fatigue and a full stomach. Based on the assumption that natural daily feeding movements comprise the cycle of chopsticks or a spoon traveling to and from the bowl and the mouth, starting and ending postures were not predefined to capture these natural cyclic daily movement data. The initial measurement position is illustrated in Figure 2.

### Measurement Instruments and Data Processing

Kinematic data at a frequency of 120 Hz were obtained using a 3D motion analysis system based on 17 inertial sensor units (Xsens MVN system, Xsens Technologies BV). Validation studies for this system have been previously conducted [15-19]. The inertial sensors were attached to participant body parts using a Lycra suit, gloves, and a headband and were confirmed not to shift on the body surface or interfere with utensils and movements during feeding. The present tasks and measurements were valid; however, they might not be confirmed as sensor slipping or skin artifacts and interference with objects or bodies are recognized sources of error that could compromise the accuracy of joint angle and position data. The biomechanical model of the system consisted of 23 body parts, including the head, neck, 6 vertebrae, pelvis, scapulae, upper arms, forearms, hands, thighs, tibiae, feet, and toes. Joint angles were calculated based on the positions and orientations of the body parts. The neutral joint angle position of the model corresponded to a standing posture with the upper limbs alongside the trunk, the face directed forward, feet parallel at a distance of 1-foot width, and palms facing forward.

When the participants ate the food 3 times sequentially, there were 2 eating cycles. One success cycle of these 2 eating cycles (or the first cycle if both were successful) in each condition was selected for each participant for analysis. The eating cycle was defined as the interval between the moment after the utensil left the mouth and the moment immediately before it next left the mouth. This was confirmed using synchronized video recordings. To simplify the analysis, failure cycles involving the following were excluded: excessive upper limb elevation during food transport, looking away from the bowl or food, extraneous head movements, separating food, or taking more than 1 scoop of yogurt. Considering performing analysis and maintaining sample size, we decided to focus on analyzing 1 successful eating cycle, which was defined as a natural attempt of daily movements, of 2 cycles regarding each condition in each participant, excluding failed cycles that sometimes were observed.

Pauses during the reaching phase (observed in 84% of chopstick tasks and 65% of spoon tasks in the final analysis) and attempts to pick up the pickles more than once (28%) were frequently observed but not excluded from the analysis. Only participants with complete task data were included in the final analysis.

**Figure 3.**
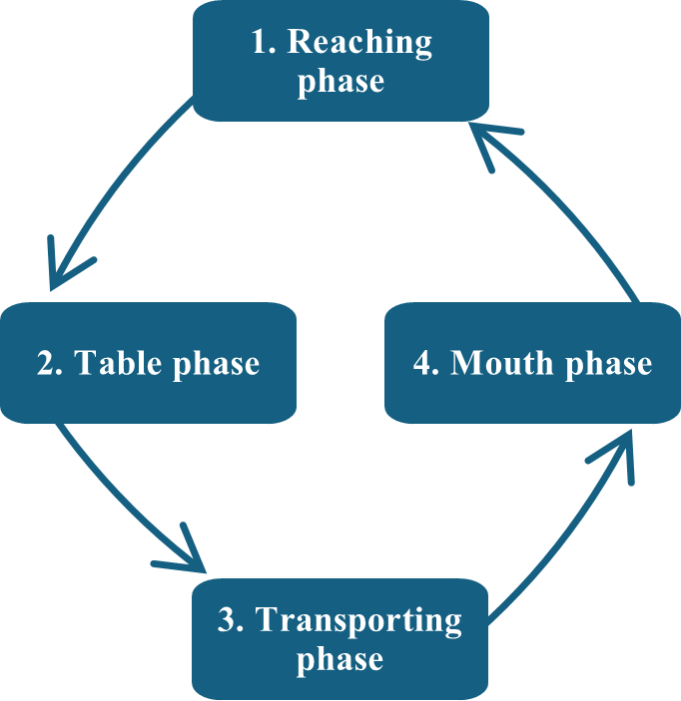
One eating cycle contains 4 phases. The arrows indicate the order of eating phase. The reaching phase is when the utensils move from the mouth to the food. The table phase is when the food is picked up or spooned up. The transporting phase is when the food moves from the bowl on the table to the mouth. The mouth phase is when the utensils are in the mouth.

One eating cycle was divided into the following four phases: (1) reaching phase—from the moment the utensil left the mouth to the point of reaching the food, (2) table phase—picking up or scooping the food, (3) transporting phase—moving the food to the mouth, and (4) mouth phase—inserting the food into the mouth (Figure 3). These phases were defined because upper body joint motions differ during each feeding phase when using a spoon [5], which was expected to apply to chopstick use as well.

Joint kinematic data from the biomechanical model were analyzed, including right shoulder flexion, abduction, and internal rotation; right elbow flexion and pronation; right wrist dorsiflexion and ulnar deviation (palmar flexion and radial deviation in the model); neck flexion, right lateral flexion, and right rotation (left rotation in the model); and right hip flexion. These joint kinematics were summarized as the maximum and minimum joint angles and ranges of angle change in each feeding phase. Movement time(s) was calculated in all phases.

The right-hand spatiotemporal parameters during the transporting phase included the actual distance traveled (meters), relative distance traveled, and maximum velocity (meters per second). The timing of maximum velocity (%) was calculated as motor control strategy metrics. The number of movement units was used to measure hand movement smoothness. The origin point for the right hand was defined as the midpoint between the styloid processes. A movement unit was defined as the difference between a local minimum and the next maximum velocity value that exceeded an amplitude limit of 20 millimeters per second, with a minimum interval of 150 milliseconds between peaks. This parameter was adapted from a task originally defined for drinking motions [20]. Most spatiotemporal parameters were limited to the transporting phase, as the start and end points of the reaching movement were clearly defined and undisturbed. Movements during the reaching phase showed frequent disruptions, and those during the table and mouth phases were not reaching movements. These hand spatiotemporal parameters could not be interpreted according to the common concept of reaching movement parameters [13,14] and, consequently, were excluded from analysis.

### Data Analysis

All metrics were analyzed separately for each sex because of known differences in daily movements [8,9,21-24] and fit to clinical assessments practically. The data are displayed as medians with IQRs. Since the study design compared movements between eating solid foods with chopsticks and semisolid foods with a spoon, these 2 task conditions were compared using the Wilcoxon signed rank test using IBM SPSS for Windows (version 27.0; IBM Corp), with a significance level of *P*<.05. Effect sizes (*r*) were calculated and categorized as small (|0.3|), medium (|0.5|), or large (|0.6|) [25].

### Ethical Considerations

The protocol for this cross-sectional study, including informed consent using an opt-out method, was approved by the research ethics committee of the Faculty of Medicine of the University of Miyazaki (Miyazaki-shi, Japan; approval number O-1501). Since the sample data were derived from past studies [4,5] and participants could not be contacted directly, information related to this study was published on the local institutional website, giving past participants the opportunity to opt out of the study at any time. All participants provided written informed consent prior to participation in the previous studies [4,5]. The data were anonymized in both the previous and the present studies.

## Results

### Overview

Forty-three participants with 1 success cycle regarding each condition have been selected, excluding 7 participants with 2 failed cycles regarding 1 of 2 conditions. The analyzed study sample included 22 male and 21 female participants. The mean (SD) age and height of the male participants were 27.9 (5.5) years and 172.0 (5.1) cm, respectively, while those of the female participants were 26.9 (4.7) years and 158.0 (4.4) cm, respectively.

### Joint Angles During Feeding Phases

In male participants (Table 1), various maximum and minimum joint angles, including shoulder flexion, abduction, and internal rotation; wrist ulnar deviation; and neck right lateral flexion, were lower when using chopsticks compared with when using spoons across many feeding phases. Additionally, during the reaching phase, the minimum wrist dorsiflexion angle with chopsticks was smaller than that with the spoon. In contrast, most elbow flexion and some neck right rotation angles were greater with chopsticks than with the spoon.

**Table 1.** Comparison of maximum and minimum joint angles between chopstick use for eating solid foods and spoon use for eating semisolid foods during each feeding phase in male participants^a^.

Motion direction and Phase	Maximum joint angle (degrees)					Minimum joint angle (degrees)				
	Chopsticks, median (IQR)	Spoon, median (IQR)	*z* value	*P* value	*r* value	Chopsticks, median (IQR)	Spoon, median (IQR)	*z* value	*P* value	*r* value
Shoulder flexion										
Re	**23 (21 to 30**)	**36 (31 to 42)** ^**b**^	−3.945	<.001	−0.595	**16 (13 to 20**)	**19 (16 to 24)^b^**	−3.1	.002	−0.467
Ta	**21 (16 to 27**)	**23 (19 to 31)^b^**	−2.029	.04	−0.306	19 (14 to 26)	22 (19 to 27)	−1.737	.08	−0.262
Tr	**25 (19 to 31**)	**36 (31 to 45)** ^**b**^	−4.01	<.001	−0.605	**18 (15 to 24**)	**23 (19 to 30)^b^**	−3.1	.002	−0.467
Mo	**25 (19 to 32**)	**41 (33 to 49)** ^**b**^	−4.042	<.001	−0.609	**23 (17 to 29**)	**36 (29 to 44)^b^**	−4.107	<.001	−0.619
Shoulder abduction										
Re	**32 (27 to 38**)	**38 (33 to 47)** ^**b**^	−3.685	<.001	−0.556	**24 (20 to 29**)	**28 (22 to 33)^b^**	−2.808	.005	−0.423
Ta	30 (24 to 35)	31 (24 to 39)	−1.737	.08	−0.262	30 (24 to 34)	30 (23 to 36)	−1.315	.19	−0.198
Tr	**32 (27 to 40**)	**37 (32 to 48)^b^**	−3.847	<.001	−0.58	28 (24 to 34)	31 (24 to 39)	−1.672	.10	−0.252
Mo	**32 (26 to 40**)	**38 (33 to 48)^b^**	−3.847	<.001	−0.58	**31 (26 to 39**)	**36 (31 to 47)^b^**	−3.912	<.001	−0.59
Shoulder internal rotation										
Re	**0 (−6 to 8**)	**2 (−1 to 15)^b^**	−3.1	.002	−0.467	**−12 (−14 to −3**)	**−7 (−10 to 4)^b^**	−3.36	.001	−0.507
Ta	**−1 (−6 to 11**)	**4 (−1 to 11)^b^**	−3.036	.002	−0.458	**−4 (−8 to 5**)	**1 (−3 to 9)^b^**	−2.646	.01	−0.399
Tr	**−3 (−6 to 8**)	**4 (0 to 16)^b^**	−3.912	<.001	−0.59	**−8 (−10 to 2**)	**1 (−5 to 8)^b^**	−3.847	<.001	−0.58
Mo	**−5 (−10 to 3**)	**6 (−1 to 15)^b^**	−3.977	<.001	−0.6	**−8 (−11 to 1**)	**1 (−5 to 13)^b^**	−4.074	<.001	−0.614
Elbow flexion										
Re	**125 (117 to 132)^b^**	**121 (114 to 129**)	−3.36	.001	−0.507	97 (88 to 106)	100 (93 to 109)	−1.51	.13	−0.228
Ta	102 (97 to 112)	105 (98 to 114)	−0.568	.57	−0.086	**99 (89 to 107)^b^**	**98 (88 to 105**)	−2.062	.04	−0.311
Tr	**126 (118 to 133)^b^**	**120 (115 to 129**)	−3.393	.001	−0.512	**101 (91 to 108)^b^**	**99 (84 to 107**)	−2.451	.01	−0.37
Mo	**131 (120 to 136)^b^**	**127 (118 to 133**)	−3.295	.001	−0.497	**125 (115 to 132)^b^**	**119 (113 to 127**)	−3.523	<.001	−0.531
Forearm pronation										
Re	90 (83 to 101)	99 (86 to 110)	−1.607	.11	−0.242	**26 (18 to 32**)	**34 (24 to 39)^b^**	−2.711	.007	−0.409
Ta	**90 (80 to 101**)	**99 (86 to 108)^b^**	−3.165	.002	−0.477	**77 (73 to 89)^b^**	**75 (55 to 83**)	−2.289	.02	−0.345
Tr	**78 (73 to 88)^b^**	**74 (55 to 83**)	−2.289	.02	−0.345	31 (23 to 37)	32 (23 to 38)	−0.828	.41	−0.125
Mo	31 (23 to 37)	34 (25 to 39)	−0.536	.59	−0.081	22 (16 to 29)	24 (16 to 32)	−0.016	.99	−0.002
Wrist dorsal flexion										
Re	26 (22 to 43)	29 (21 to 42)	−0.243	.81	−0.037	**13 (2 to 24**)	**20 (8 to 34)^b^**	−2.289	.02	−0.345
Ta	22 (9 to 37)	28 (16 to 38)	−1.217	.22	−0.183	16 (4 to 25)	21 (12 to 34)	−1.542	.12	−0.232
Tr	26 (15 to 36)	27 (18 to 39)	−0.243	.81	−0.037	19 (7 to 30)	20 (11 to 31)	−0.925	.36	−0.139
Mo	22 (11 to 33)	23 (11 to 31)	−0.016	.99	−0.002	20 (9 to 30)	20 (7 to 29)	−0.146	.88	−0.022
Wrist ulnar deviation										
Re	**5 (−3 to 10**)	**11 (4 to 16)^b^**	−3.587	<.001	−0.541	−7 (−14 to 2)	−5 (−14 to 5)	−1.347	.18	−0.203
Ta	**1 (−5 to 9**)	**7 (3 to 13)^b^**	−3.328	.001	−0.502	−2 (−10 to 7)	−4 (−12 to 5)	−0.86	.39	−0.13
Tr	**3 (−3 to 12**)	**12 (5 to 16)^b^**	−3.977	<.001	−0.6	**−1 (−7 to 5**)	**5 (1 to 12)^b^**	−3.782	<.001	−0.57
Mo	**2 (−4 to 11**)	**13 (3 to 18)^b^**	−4.074	<.001	−0.614	**1 (−5 to 8**)	**10 (3 to 15)^b^**	−4.042	<.001	−0.609
Neck flexion										
Re	27 (22 to 29)	26 (22 to 28)	−1.445	.15	−0.218	19 (14 to 25)	21 (18 to 26)	−1.25	.21	−0.188
Ta	26 (22 to 30)	25 (23 to 28)	−0.373	.71	−0.056	25 (21 to 29)	24 (22 to 27)	−0.828	.41	−0.125
Tr	25 (21 to 29)	25 (22 to 27)	−0.601	.55	−0.091	21 (18 to 25)	20 (18 to 25)	−1.542	.12	−0.232
Mo	22 (19 to 26)	22 (19 to 28)	−0.73	.47	−0.11	21 (18 to 24)	18 (17 to 24)	−1.899	.06	−0.286
Neck right lateral flexion										
Re	**−1 (−2 to 0**)	**3 (2 to 4)^b^**	−4.107	<.001	−0.619	**−4 (-5 to −3**)	**−2 (−4 to 0)^b^**	−3.036	.002	−0.458
Ta	**−3 (−4 to −2**)	**−2 (−3 to 0)^b^**	−3.036	.002	−0.458	**−3 (−5 to −2**)	**−2 (−4 to 0)^b^**	−2.678	.007	−0.404
Tr	**−2 (-3 to −1**)	**2 (−1 to 3)^b^**	−3.945	<.001	−0.595	**−3 (−4 to −3**)	**−2 (−3 to 0)^b^**	−3.393	.001	−0.512
Mo	**−2 (−3 to 0**)	**4 (2 to 5)^b^**	−4.107	<.001	−0.619	**−3 (-4 to −1**)	**2 (−1 to 3)^b^**	−3.977	<.001	−0.6
Neck right rotation										
Re	**4 (2 to 6)^b^**	**2 (1 to 3**)	−3.263	.001	−0.492	0 (−1 to 2)	0 (−1 to 2)	−0.211	.83	−0.032
Ta	1 (0 to 2)	1 (0 to 2)	−0.016	.99	−0.002	1 (−1 to 2)	1 (−1 to 1)	−0.179	.86	−0.027
Tr	**4 (1 to 6)^b^**	**3 (0 to 3**)	−3.165	.002	−0.477	1 (−1 to 2)	1 (−1 to 2)	−0.146	.88	−0.022
Mo	**5 (3 to 7)^b^**	**3 (2 to 5**)	−3.393	.001	−0.512	**4 (1 to 5)^b^**	**2 (0 to 3**)	−3.068	.002	−0.463
Hip flexion										
Re	51 (44 to 57)	50 (45 to 54)	−0.73	.47	−0.11	43 (37 to 51)	44 (38 to 50)	−0.016	.99	−0.002
Ta	44 (38 to 51)	46 (38 to 51)	−0.341	.73	−0.051	44 (37 to 51)	45 (38 to 51)	−0.373	.71	−0.056
Tr	50 (43 to 58)	50 (44 to 54)	−0.763	.45	−0.115	44 (38 to 51)	46 (38 to 51)	−0.438	.66	−0.066
Mo	51 (45 to 58)	51 (46 to 56)	−0.179	.86	−0.027	50 (43 to 56)	51 (44 to 54)	−0.308	.76	−0.046

a Significant differences in each phase, as determined using the Wilcoxon signed rank test (*P*<.05), are shown as values in boldface. "*r*" denotes the effect size. Each feeding phase shows the reaching phase as “Re,” the table phase as “Ta,” the transporting phase as “Tr,” and the mouth phase as “Mo.”

b Larger values from comparisons between chopstick and spoon conditions.

Forearm pronation showed differing trends according to the feeding phase. The minimum forearm pronation angle during the reaching phase and maximum angle during the table phase were smaller with chopsticks than with the spoon. Conversely, the minimum angle during the table phase and maximum angle during the transporting phase were larger with chopsticks. The differences between utensils showed small to large effect sizes. Neck and hip flexion angles did not differ significantly between utensils.

In female participants (Table 2), all shoulder joint angles, nearly all wrist dorsiflexion angles, and neck right lateral flexion angles were smaller with chopsticks than with the spoon. In contrast, most elbow flexion angles were larger when using chopsticks. Forearm pronation and wrist ulnar deviation angles varied inconsistently across feeding phases. In half the forearm pronation angles, chopsticks showed smaller values than did the spoon. However, the minimum angle during the table phase and maximum angle during the transporting phase were larger in the chopstick condition. Minimum wrist ulnar deviation angles during the reaching and table phases were larger with chopsticks, while other angles were smaller. These differences showed small to large effect sizes. Most neck flexion angles and all neck right rotation and hip flexion angles were comparable between chopsticks and spoons.

**Table 2.** Comparison of maximum and minimum joint angles between chopstick use for eating solid foods and spoon use for eating semisolid foods during each feeding phase in female participants^a^.

Motion direction and Phase	Maximum joint angle (degrees)					Minimum joint angle (degrees)				
	Chopsticks, median (IQR)	Spoon, median (IQR)	*z* value	*P* value	*r* value	Chopsticks, median (IQR)	Spoon, median (IQR)	*z* value	*P* value	*r* value
Shoulder flexion										
Re	**20 (14 to 27**)	**37 (31 to 44)^b^**	-4.015	<.001	–0.62	**10 (4 to 12**)	**10 (6 to 22)^b^**	–2.45	.01	–0.378
Ta	**14 (7 to 18**)	**15 (10 to 25)^b^**	–2.694	.007	–0.416	**14 (6 to 17**)	**13 (7 to 23)^b^**	–2.485	.01	–0.383
Tr	**21 (12 to 28**)	**37 (31 to 44)^b^**	–4.015	<.001	–0.62	**13 (6 to 17**)	**14 (9 to 24)^b^**	–2.972	.003	–0.459
Mo	**22 (18 to 31**)	**40 (37 to 49)^b^**	–4.015	<.001	–0.62	**20 (15 to 27**)	**37 (31 to 44)^b^**	–4.015	<.001	–0.62
Shoulder abduction										
Re	**31 (28 to 34**)	**37 (32 to 45)^b^**	–3.98	<.001	–0.614	**21 (19 to 24**)	**23 (19 to 28)^b^**	–2.589	.01	–0.399
Ta	**24 (22 to 28**)	**28 (24 to 36)^b^**	–3.285	.001	–0.507	**23 (21 to 27**)	**28 (23 to 33)^b^**	–3.319	.001	–0.512
Tr	**30 (27 to 35**)	**38 (33 to 47)^b^**	–4.015	<.001	–0.62	**24 (21 to 28**)	**28 (24 to 33)^b^**	–3.493	<.001	–0.539
Mo	**29 (27 to 36**)	**39 (32 to 48)^b^**	–3.98	<.001	–0.614	**28 (26 to 32**)	**36 (31 to 45)^b^**	–3.945	<.001	–0.609
Shoulder internal rotation										
Re	**3 (0 to 8**)	**13 (10 to 18)^b^**	–3.98	<.001	–0.614	**-5 (-8 to ‐1**)	**5 (-4 to 7)^b^**	–3.493	<.001	–0.539
Ta	**3 (0 to 8**)	**11 (6 to 15)^b^**	–3.25	.001	–0.501	**1 (-2 to 5**)	**9 (5 to 13)^b^**	–3.041	.002	–0.469
Tr	**3 (0 to 6**)	**13 (9 to 17)^b^**	–4.015	<.001	–0.62	**-1 (-5 to 0**)	**7 (2 to 11)^b^**	–3.875	<.001	–0.598
Mo	**4 (-1 to 8**)	**15 (10 to 21)^b^**	–4.015	<.001	–0.62	**0 (-3 to 5**)	**13 (6 to 15)^b^**	–4.015	<.001	–0.62
Elbow flexion										
Re	**135 (130 to 138)^b^**	**128 (120 to 134**)	–3.841	<.001	–0.593	106 (101 to 111)	103 (97 to 112)	–0.504	.61	–0.078
Ta	106 (104 to 111)	103 (97 to 112)	–1.095	.27	–0.169	**103 (97 to 110)^b^**	**97 (90 to 104**)	–3.041	.002	–0.469
Tr	**134 (125 to 137)^b^**	**128 (122 to 131**)	–3.076	.002	–0.475	**104 (97 to 111)^b^**	**97 (90 to 106**)	–3.389	.001	–0.523
Mo	**138 (131 to 141)^b^**	**131 (127 to 137**)	–3.041	.002	–0.469	**134 (126 to 138)^b^**	**127 (120 to 131**)	–3.736	<.001	–0.576
Forearm pronation										
Re	**102 (80 to 116**)	**109 (99 to 123)^b^**	–2.763	.01	–0.426	**17 (12 to 43**)	**32 (19 to 49)^b^**	–3.285	.001	–0.507
Ta	**102 (81 to 119**)	**111 (99 to 122)^b^**	–2.728	.01	–0.421	**93 (68 to 108)^b^**	**80 (64 to 91**)	–3.319	.001	–0.512
Tr	**93 (68 to 107)^b^**	**80 (64 to 91**)	–3.215	.001	–0.496	26 (16 to 44)	29 (18 to 45)	–0.122	.90	–0.019
Mo	**26 (15 to 41**)	**31 (20 to 48)^b^**	–2.346	.02	–0.362	17 (10 to 35)	27 (11 to 40)	–1.199	.23	–0.185
Wrist dorsal flexion										
Re	**22 (15 to 37**)	**34 (27 to 40)^b^**	–3.563	<.001	–0.55	**9 (-1 to 21**)	**20 (14 to 31)^b^**	–3.875	<.001	–0.598
Ta	**23 (12 to 34**)	**31 (21 to 37)^b^**	–3.18	.001	–0.491	**18 (3 to 25**)	**23 (15 to 31)^b^**	–2.694	.007	–0.416
Tr	**25 (15 to 37**)	**31 (23 to 39)^b^**	–2.381	.02	–0.367	17 (7 to 30)	23 (11 to 31)	–1.616	.11	–0.249
Mo	18 (10 to 34)	25 (15 to 33)	–1.929	.054	–0.298	**17 (7 to 31**)	**22 (12 to 31)^b^**	–2.033	.04	–0.314
Wrist ulnar deviation										
Re	**12 (5 to 17**)	**14 (9 to 18)^b^**	–2.381	.02	–0.367	**4 (-2 to 10)^b^**	**-1 (-10 to 4**)	–3.319	.001	–0.512
Ta	**10 (3 to 15**)	**10 (6 to 18)^b^**	–1.964	.05	–0.303	**6 (0 to 13)^b^**	**-3 (-11 to 4**)	–3.91	<.001	–0.603
Tr	**12 (5 to 15**)	**15 (9 to 19)^b^**	–3.424	.001	–0.528	**9 (1 to 13**)	**10 (5 to 14)^b^**	–2.346	.02	–0.362
Mo	**10 (6 to 16**)	**15 (9 to 20)^b^**	–3.806	<.001	–0.587	**9 (5 to 13**)	**12 (9 to 19)^b^**	–3.597	<.001	–0.555
Neck flexion										
Re	27 (25 to 30)	27 (25 to 29)	–1.199	.23	–0.185	19 (10 to 25)	18 (15 to 23)	–0.678	.498	–0.105
Ta	27 (25 to 30)	27 (25 to 29)	–0.33	.74	–0.051	26 (24 to 29)	26 (25 to 28)	–0.226	.82	–0.035
Tr	26 (24 to 29)	27 (25 to 28)	–0.539	.59	–0.083	18 (11 to 24)	17 (12 to 19)	–1.929	.054	–0.298
Mo	20 (11 to 25)	20 (15 to 22)	–0.678	.50	–0.105	**18 (9 to 23)^b^**	**14 (11 to 18**)	–2.485	.01	–0.383
Neck right lateral flexion										
Re	**2 (–2 to 3**)	**3 (2 to 6)^b^**	–3.736	<.001	–0.576	**-2 (-4 to 0**)	**-1 (-2 to 0)^b^**	–2.52	.01	–0.389
Ta	**–1 (–3 to 2**)	**0 (-1 to 2)^b^**	–2.242	.03	–0.346	–2 (–3 to 1)	-1 (-2 to 0)	–1.825	.07	–0.282
Tr	**2 (–1 to 3**)	**3 (1 to 5)^b^**	–3.667	<.001	–0.566	**–1 (–3 to 1**)	**0 (–1 to 1)^b^**	–3.007	.003	–0.464
Mo	**1 (–1 to 3**)	**4 (3 to 7)^b^**	–3.493	<.001	–0.539	**1 (–2 to 3**)	**2 (1 to 5)^b^**	–3.563	<.001	–0.55
Neck right rotation										
Re	3 (1 to 5)	3 (1 to 5)	–1.025	.31	–0.158	0 (–2 to 1)	0 (–2 to 0)	–0.295	.77	–0.046
Ta	1 (–1 to 1)	0 (–2 to 1)	–0.017	.99	–0.003	0 (–1 to 1)	0 (–2 to 1)	–0.226	0.82	–0.035
Tr	3 (1 to 4)	3 (2 to 4)	–0.469	.64	–0.072	1 (–1 to 1)	0 (–2 to 1)	–0.052	.96	–0.008
Mo	4 (2 to 6)	5 (2 to 6)	–0.226	.82	–0.035	3 (1 to 4)	3 (0 to 4)	–0.608	.54	–0.094
Hip flexion										
Re	52 (42 to 60)	52 (42 to 58)	–1.095	.27	–0.169	44 (38 to 53)	45 (38 to 52)	–0.052	.96	–0.008
Ta	45 (39 to 54)	45 (38 to 53)	–0.956	.34	–0.148	44 (38 to 54)	44 (38 to 52)	–0.504	.61	–0.078
Tr	53 (43 to 58)	49 (42 to 57)	–1.025	.31	–0.158	45 (39 to 54)	44 (38 to 53)	–1.13	.26	–0.174
Mo	54 (44 to 60)	53 (43 to 58)	–1.06	.29	–0.164	53 (43 to 58)	49 (42 to 57)	–1.616	.11	–0.249

a Significant differences in each phase, as determined using the Wilcoxon signed rank test (*P*<.05), are shown as values in boldface. "*r*" denotes the effect size. Each feeding phase shows the reaching phase as “Re,” the table phase as “Ta,” the transporting phase as “Tr,” and the mouth phase as “Mo.”

b Larger values in comparisons between chopstick and spoon conditions.

### Changes in Joint Angles

In male participants (Table 3), changes in shoulder flexion, abduction, and neck right lateral flexion angles were generally smaller in the chopstick condition than in the spoon condition across all feeding phases. Additionally, wrist ulnar deviation, neck flexion, and hip flexion showed smaller changes in some phases when using chopsticks. Conversely, neck right rotation in most phases and wrist dorsiflexion during the reaching phase showed increased changes with chopsticks. Elbow flexion showed greater changes in the reaching phase when using chopsticks; however, the changes were smaller in the table and mouth phases. Changes in forearm pronation were smaller during the table phase but larger during the transporting phase. Shoulder internal rotation changes across all phases showed no significant differences between utensils. Effect sizes for these differences were small to large.

**Table 3.** Comparison of joint angle changes between chopstick use for eating solid foods and spoon use for eating semisolid foods during each feeding phase in male participants^a^.

Motion direction and Phases	Joint angle change (degrees)				
	Chopsticks, median (IQR)	Spoon, median (IQR)	*z* value	*P* value	*r* value
Shoulder flexion					
Re	**7 (5 to 12**)	**18 (11 to 21)^b^**	–3.62	<.001	–0.546
Ta	1 (1 to 3)	2 (1 to 3)	–1.217	.22	–0.183
Tr	**6 (4 to 9**)	**13 (7 to 18)^b^**	–3.425	.001	–0.516
Mo	**2 (2 to 3**)	**3 (3 to 5)^b^**	–2.841	.01	–0.428
Shoulder abduction					
Re	**6 (4 to 12**)	**11 (7 to 13)^b^**	–2.419	.02	–0.365
Ta	**1 (0 to 1**)	**1 (1 to 2)^b^**	–2.808	.005	–0.423
Tr	**4 (2 to 6**)	**7 (5 to 11)^b^**	–3.685	<.001	–0.556
Mo	**1 (1 to 1**)	**2 (1 to 2)^b^**	–2.646	.008	–0.399
Shoulder internal rotation					
Re	9 (6 to 15)	9 (6 to 14)	–1.25	.21	–0.188
Ta	2 (1 to 4)	3 (2 to 4)	–0.958	.34	–0.144
Tr	4 (2 to 8)	4 (2 to 8)	–0.308	.76	–0.046
Mo	2 (1 to 4)	3 (2 to 4)	–0.828	.41	–0.125
Elbow flexion					
Re	**26 (22 to 36)^b^**	**22 (13 to 25**)	–3.036	.002	–0.458
Ta	**2 (1 to 4**)	**7 (4 to 13)^b^**	–3.555	<.001	–0.536
Tr	25 (21 to 30)	23 (20 to 28)	–1.055	.29	–0.159
Mo	**4 (3 to 5**)	**6 (4 to 7)^b^**	–2.354	.02	–0.355
Forearm pronation					
Re	68 (57 to 76)	67 (60 to 77)	–0.049	.96	–0.007
Ta	**11 (7 to 13**)	**26 (22 to 34)^b^**	–4.074	<.001	–0.614
Tr	**50 (44 to 55)^b^**	**42 (34 to 52**)	–2.484	.01	–0.374
Mo	8 (5 to 11)	9 (8 to 11)	–1.704	.09	–0.257
Wrist dorsal flexion					
Re	**16 (8 to 29)^b^**	**10 (7 to 14**)	–2.289	.02	–0.345
Ta	6 (4 to 9)	5 (3 to 7)	–1.412	.16	–0.213
Tr	8 (5 to 11)	4 (3 to 8)	–1.899	.06	–0.286
Mo	2 (1 to 4)	2 (1 to 3)	–0.601	.55	–0.091
Wrist ulnar deviation					
Re	**9 (5 to 14**)	**15 (9 to 22)^b^**	–2.386	.02	–0.36
Ta	**4 (2 to 6**)	**11 (6 to 18)^b^**	–3.88	<.001	–0.585
Tr	4 (3 to 6)	4 (2 to 7)	–0.86	.39	–0.13
Mo	2 (1 to 2)	1 (1 to 2)	–0.925	.36	–0.139
Neck flexion					
Re	4 (2 to 9)	3 (2 to 6)	–1.769	.08	–0.267
Ta	0 (0 to 1)	1 (0 to 1)	–1.088	.28	–0.164
Tr	3 (1 to 6)	4 (2 to 6)	–0.99	.32	–0.149
Mo	**1 (1 to 2**)	**3 (2 to 4)^b^**	–3.1	.002	–0.467
Neck right lateral flexion					
Re	**2 (2 to 4**)	**5 (4 to 7)^b^**	–4.107	<.001	–0.619
Ta	0 (0 to 1)	1 (0 to 1)	–1.347	.18	–0.203
Tr	**1 (1 to 3**)	**2 (1 to 5)^b^**	–2.516	.01	–0.379
Mo	**1 (0 to 1**)	**2 (2 to 3)^b^**	–3.945	<.001	–0.595
Neck right rotation					
Re	**3 (2 to 5)^b^**	**2 (1 to 3**)	–3.717	<.001	–0.56
Ta	0 (0 to 1)	0 (0 to 1)	–0.016	.99	–0.002
Tr	**3 (1 to 4)^b^**	**1 (1 to 2**)	–2.971	.003	–0.448
Mo	**2 (1 to 3)^b^**	**2 (1 to 2**)	–2.354	.02	–0.355
Hip flexion					
Re	7 (3 to 9)	6 (3 to 8)	–1.412	.16	–0.213
Ta	**0 (0 to 0**)	**0 (0 to 1)^b^**	–2.354	.02	–0.355
Tr	5 (2 to 8)	5 (1 to 6)	–1.282	.20	–0.193
Mo	1 (1 to 1)	1 (0 to 2)	–0.308	.76	–0.046

a Significant differences in each phase, as determined using the Wilcoxon signed rank test (*P*<.05), are shown as values in boldface. “*r*" denotes the effect size. Each feeding phase shows the reaching phase as “Re,” the table phase as “Ta,” the transporting phase as “Tr,” and the mouth phase as “Mo.”

b Larger values in comparisons between chopstick and spoon conditions.

In female participants (Table 4), changes in shoulder flexion, abduction, and neck right lateral flexion angles across most feeding phases were smaller with chopsticks than with a spoon. Additionally, changes in shoulder internal rotation, wrist ulnar deviation, and neck and hip flexion were smaller in some phases when using chopsticks. Elbow flexion changes during the reaching phase and forearm pronation changes during the transporting phase were larger in the chopstick condition, contrasting with smaller changes in elbow flexion during the table and mouth phases and smaller forearm pronation changes during the table phase. Wrist dorsiflexion and neck right rotation changes were similar across all feeding phases. Significant differences were found in small to large effect sizes.

**Table 4.** Comparison of joint angle changes between chopstick use for eating solid foods and spoon use for eating semisolid foods during each feeding phase in female participants^a^.

Motion direction and Phases	Joint angle change (degrees)				
	Chopsticks, median (IQR)	Spoon, median (IQR)	*z* value	*P* value	*r* value
Shoulder flexion					
Re	**11 (7 to 17**)	**25 (15 to 33)^b^**	–3.98	<.001	–0.614
Ta	**1 (1 to 1**)	**2 (1 to 3)^b^**	–2.485	.01	–0.383
Tr	**7 (5 to 13**)	**19 (15 to 28)^b^**	–3.875	<.001	–0.598
Mo	**3 (2 to 4**)	**5 (4 to 7)^b^**	–3.528	<.001	–0.544
Shoulder abduction					
Re	**8 (6 to 14**)	**13 (6 to 18)^b^**	–2.624	.01	–0.405
Ta	1 (0 to 2)	1 (1 to 2)	–1.581	.11	–0.244
Tr	**5 (3 to 8**)	**8 (5 to 17)^b^**	–2.589	.01	–0.399
Mo	**1 (1 to 2**)	**2 (1 to 3)^b^**	–2.45	.01	–0.378
Shoulder internal rotation					
Re	9 (6 to 12)	9 (6 to 16)	–1.651	.10	–0.255
Ta	**1 (1 to 2**)	**3 (2 to 3)^b^**	to 2.311	.02	–0.357
Tr	5 (3 to 6)	5 (4 to 10)	–1.894	.06	–0.292
Mo	**2 (2 to 4**)	**4 (3 to 5)^b^**	–3.076	.002	–0.475
Elbow flexion					
Re	**29 (22 to 32)^b^**	**21 (14 to 33**)	–2.172	.03	–0.335
Ta	**3 (2 to 4**)	**8 (6 to 12)^b^**	–3.702	<.001	–0.571
Tr	28 (22 to 32)	30 (24 to 34)	–0.782	.43	–0.121
Mo	**4 (3 to 6**)	**7 (5 to 8)^b^**	–3.702	<.001	–0.571
Forearm pronation					
Re	74 (63 to 87)	78 (67 to 82)	–0.678	.50	–0.105
Ta	**8 (5 to 15**)	**33 (23 to 45)^b^**	–4.015	<.001	–0.62
Tr	**58 (52 to 70)^b^**	**49 (38 to 53**)	–3.424	.001	–0.528
Mo	7 (3 to 11)	10 (6 to 12)	–1.616	.11	–0.249
Wrist dorsal flexion					
Re	12 (8 to 20)	11 (6 to 16)	–1.86	.06	–0.287
Ta	8 (5 to 12)	8 (3 to 13)	–0.504	.61	–0.078
Tr	5 (3 to 7)	7 (4 to 11)	–1.825	.07	–0.282
Mo	3 (2 to 4)	3 (2 to 3)	–1.13	.26	–0.174
Wrist ulnar deviation					
Re	**7 (3 to 9**)	**14 (10 to 25)^b^**	–3.354	.001	–0.518
Ta	**3 (1 to 3**)	**14 (6 to 24)^b^**	–4.015	<.001	–0.62
Tr	4 (3 to 5)	5 (3 to 6)	–1.269	.21	–0.196
Mo	1 (1 to 2)	1 (1 to 3)	–1.442	.15	–0.223
Neck flexion					
Re	8 (3 to 13)	7 (5 to 10)	–1.408	.16	–0.217
Ta	1 (0 to 1)	1 (0 to 1)	–0.365	.72	–0.056
Tr	**8 (3 to 11**)	**10 (8 to 13)^b^**	–2.416	.02	–0.373
Mo	**2 (1 to -2**)	**3 (2 to 5)^b^**	–3.98	<.001	–0.614
Neck right lateral flexion					
Re	**2 (1 to 3**)	**4 (2 to 8)^b^**	–3.215	.001	–0.496
Ta	**0 (0 to 1**)	**1 (0 to 1)^b^**	–1.964	.05	–0.303
Tr	**2 (1 to 3**)	**3 (1 to 6)^b^**	–2.589	.01	–0.399
Mo	**1 (1 to 1**)	**2 (1 to 2)^b^**	–3.18	.001	–0.491
Neck right rotation					
Re	4 (2 to 5)	3 (1 to 5)	–1.303	.19	–0.201
Ta	0 (0 to 1)	0 (0 to 1)	–1.442	.15	–0.223
Tr	3 (2 to 4)	3 (2 to 4)	–0.956	.34	–0.148
Mo	2 (1 to 2)	2 (1 to 2)	–0.713	.48	–0.11
Hip flexion					
Re	6 (5 to 8)	4 (2 to 7)	–1.408	.16	–0.217
Ta	0 (0 to 0)	0 (0 to 0)	–1.512	.13	–0.233
Tr	4 (2 to 7)	4 (1 to 7)	–0.365	.72	–0.056
Mo	**1 (0 to 1**)	**2 (1 to 2)^b^**	–3.076	.002	–0.475

a Significant differences in each phase, as determined using the Wilcoxon signed rank test (*P*<.05), are shown as values in boldface. "*r*" denotes the effect size. Each feeding phase shows the reaching phase as “Re,” the table phase as “Ta,” the transporting phase as “Tr,” and the mouth phase as “Mo.”

b Larger values in comparisons between chopstick and spoon conditions.

### Hand Spatiotemporal Parameters

In both sexes (Table 5 for males and Table 6 for females), the reaching phase duration was longer with chopsticks than with the spoon. In contrast, the mouth phase duration was shorter with chopsticks. Additionally, female participants showed a shorter transporting phase duration when using chopsticks. These differences showed small to large effect sizes.

**Table 5.** Comparison of movement times between chopstick use for eating solid foods and spoon use for eating semisolid foods during each feeding phase in male participants^a^.

Variable and Phase	Chopsticks, median (IQR)	Spoon, median (IQR)	*z* value	*P* value	*r* value
Movement time (seconds)					
Re	**4.9 (2.4 to 9.6)^b^**	**2.9 (1.8 to 3.5**)	–3.393	.001	–0.512
Ta	1 (0.7 to 2.2)	1 (0.7 to 1.4)	–0.438	.66	–0.066
Tr	0.8 (0.6 to 1.4)	0.9 (0.7 to 1.2)	–1.136	.26	–0.171
Mo	**0.4 (0.3 to 0.5**)	**0.7 (0.6 to 0.8)^b^**	–3.979	<.001	–0.600

a Significant differences in each phase, as determined using the Wilcoxon signed rank test (*P*<.05), are shown as values in boldface. "*r*" denotes the effect size. Each feeding phase shows the reaching phase as “Re,” the table phase as “Ta,” the transporting phase as “Tr,” and the mouth phase as “Mo.”

b Larger values in comparisons between chopstick and spoon conditions.

**Table 6.** Comparison of movement times between chopstick use for eating solid foods and spoon use for eating semisolid foods during each feeding phase in female participants^a^.

Variable an Phase	Chopsticks, median (IQR)	Spoon, median (IQR)	*z* value	*P *value	*r* value
Movement time (seconds)					
Re	**4.8 (3.2 to 7.3)^b^**	**2.8 (1.6 to 4.7**)	–3.702	<.001	–0.571
Ta	1.1 (0.9 to 1.8)	1.5 (1.3 to 1.7)	–0.852	.39	–0.131
Tr	**0.9 (0.8 to 1.1**)	**1.1 (1 to 1.2)^b^**	–2.103	.04	–0.324
Mo	**0.5 (0.4 to 0.7**)	**0.9 (0.8 to 1.2)^b^**	–3.98	<.001	–0.614

a Significant differences in each phase, as determined using the Wilcoxon signed rank test (*P*<.05), are shown as values in boldface. "*r*" denotes the effect size. Each feeding phase shows the reaching phase as “Re,” the table phase as “Ta,” the transporting phase as “Tr,” and the mouth phase as “Mo.”

b Larger values in comparisons between chopstick and spoon conditions.

During the transporting phase in both sexes (Table 7 for males and Table 8 for females), the actual distance traveled was shorter with chopsticks; however, the relative distance traveled was greater than that with a spoon. Velocity and maximum velocity were lower with chopsticks than with spoon. The timing of maximum velocity and the number of movement units used were comparable between utensils. Small to large effect sizes were observed for significant differences between utensil conditions.

**Table 7.** Comparison of hand spatiotemporal parameters between chopstick use for eating solid foods and spoon use for eating semisolid foods during the transporting phase in male participants^a^.

Variables	Chopsticks, median (IQR)	Spoon, median (IQR)	*z* value	*P* value	*r* value
Actual distance traveled (meters)	**0.11 (0.08 to 0.12**)	**0.14 (0.11 to 0.18)^b^**	–3.782	<.001	–0.570
Relative distance traveled	**1.17 (1.04 to 1.35)^b^**	**1.07 (1.04 to 1.11**)	–2.062	.04	–0.311
Velocity (meters per second)	**0.11 (0.08 to -0.15**)	**0.16 (0.14 to 0.19)^b^**	–3.393	.001	–0.512
Maximum velocity (meters per second)	**0.18 (0.16 to 0.28**)	**0.25 (0.22 to 0.32)^b^**	–3.068	.002	–0.463
Timing of maximum velocity (%)	70.6 (61.7 to 83.2)	60.2 (45.1 to 75.8)	–1.704	.09	–0.257
Number of movement units	5 (3.8 to 8)	4 (3 to 7)	–1.325	.19	–0.200

a Significant differences, as determined using the Wilcoxon signed rank test (*P*<.05), are shown as values in boldface. "*r*" denotes the effect size.

b Larger values in comparisons between chopstick and spoon conditions.

**Table 8.** Comparison of hand spatiotemporal parameters between chopstick use for eating solid foods and spoon use for eating semisolid foods during the transporting phase in female participants^a^.

Variables	Chopsticks, median (IQR)	Spoon, median (IQR)	*z* value	*P* value	*r* value
Actual distance traveled (meters)	**0.12 (0.11 to 0.14**)	**0.2 (0.17 to 0.22)^b^**	–3.91	<.001	–0.603
Relative distance traveled	**1.15 (1.07 to 1.2)^b^**	**1.05 (1.04 to 1.09**)	–3.424	.001	–0.528
Velocity (meters per second)	**0.12 (0.1 to 0.16**)	**0.18 (0.14 to 0.2)^b^**	–3.702	<.001	–0.571
Maximum velocity (meters per second)	**0.22 (0.19 to 0.29**)	**0.35 (0.28 to 0.37)^b^**	–3.875	<.001	–0.598
Timing of maximum velocity (%)	70.3 (63.4 to 75.9)	63.9 (56.4 to 69.2)	–1.338	.18	–0.206
Number of movement units	5 (4 to 6)	6 (4.5 to 6.5)	–1.171	.24	–0.181

a Significant differences, as determined using the Wilcoxon signed rank test (P<.05), are shown as values in boldface. "*r*" denotes the effect size.

b Larger values in comparisons between chopstick and spoon conditions.

## Discussion

### Principal Findings in Joint Kinematics

The aim of this study was to determine upper body joint angles and hand spatiotemporal parameters during the use of chopsticks for solid foods and a spoon for semisolid foods across all feeding phases by comparing these conditions. In addition to these findings observed in both sexes, using subgroup analysis, male participants demonstrated greater neck rotation motions, while female participants exhibited fewer wrist dorsiflexion angles under the chopstick condition. This comparison is crucial for clinical assessments of individuals with upper body sensorimotor dysfunctions, considering food customs in East Asia.

Various joint angle positions of the upper limb and neck were lower in the chopstick condition than in the spoon condition, except for most elbow flexion angles and some forearm pronation, wrist ulnar deviation (in females), and neck right rotation (in males) angles being higher in the chopstick condition. In addition, female participants showed smaller wrist dorsiflexion positions with chopstick use than with spoon use. However, neck and hip flexion angles were generally comparable between the conditions.

The changes in upper body joint angles across phases were smaller in the chopstick condition than in the spoon condition, except for greater or fewer changes in elbow and forearm joint angles with chopsticks, and greater wrist dorsiflexion and neck right rotation angles in male participants. Based on the comparison between the conditions, chopsticks required smaller joint angle positions and changes in shoulder and neck flexion and right lateral flexion across feeding phases and sexes.

These findings align with our hypotheses and the results from previous reports on shoulder and neck joint angles [6,12]. Chopstick use requires less control of shoulder joint motions due to how food is held compared with spoon use. This rationale is supported by the fact that chopsticks may securely hold food through a “scissor-pinching” motion, which reduces trapezius muscle activity compared with “pincer pinching” [26]. The scissor-pinching technique used with chopsticks allows for a stronger grip on the food [27]. The reduction in muscle activity may explain the smaller shoulder joint motions observed in the chopstick condition. In addition, the finding of fewer neck joint angle positions and changes in our chopstick condition is supported by findings in studies showing smaller head or trunk region motions while eating solid foods with a fork or a spoon (not-easy-to-drop-foods condition), compared with eating liquid foods with a spoon (easy-to-drop-foods condition) [8,11]. The actual differences in shoulder joint motions to control utensils and compensatory neck joint motions between chopstick use for solid foods and spoon use for semisolid foods should be clinically highlighted as normative information. However, the current experimental conditions did not clarify whether food was prone to being dropped, indicating the need for further studies.

Notably, in male participants, neck right rotation angles and angle changes during some phases were greater in the chopstick condition than in the spoon condition. Additionally, hip flexion angles and changes, which indicate trunk positioning and motion, did not differ significantly between conditions in either sex group. Proximal body part stability or movements are considered the same. Although they were not anticipated at the start of the study, these postures and movements could be important for clinical assessments. Proximal body part motions are often more pronounced when eating foods prone to being dropped [8,11], which was likely the case for the spoon condition in this study. However, this remains unclear and warrants further investigation under adjusted conditions.

Contrary to our hypothesis, motion extent at the middle and distal joints using chopsticks was not consistently reduced. For instance, when transporting food to the mouth, forearm pronation angle changes were greater in the chopstick condition than in the spoon condition. However, during the table phase (when taking food from the bowl), the changes were not significant in the chopstick condition. These joint motions are thought to be essential for approaching and manipulating food directly.

### Principal Findings in Hand Spatiotemporal Parameters

Hand spatiotemporal parameters suggest that, in contrast with spoon use, chopstick use required more time during the reaching phase and less time during the mouth phase in both sex groups, with female participants also showing shorter times during the transporting phase. These utensil and food type–related differences contradicted our hypothesis. Furthermore, this finding was not recognized in a previous study [6]. A possible reason for the contrasting results is that the previous study used pickles as the food item under both the chopstick and spoon conditions, whereas this study used pickles (solid food) with chopsticks and yogurt (semisolid food) with a spoon to align with the study aim. Yogurt may be easier to consume without chewing (requiring less time during the reaching phase) than pickles [28]. However, yogurt may be more prone to spilling than pickles (requiring more time during the transporting and mouth phases). Accordingly, the present findings related to duration differences in conditions lead to the assumption that this was due to utensil use along with respective food characteristics such as viscosity and not just dependent on utensil types.

Hand spatiotemporal parameters during the reaching movement have been reported [13,14], with clearly defined start and end positions and movement not disrupted. Accordingly, this study analyzed parameters related to distance, speed, motor control strategy, and smoothness during the transporting phase, which showed no disruption during reaching or transporting. The results were interpreted in accordance with the common concept of spatiotemporal parameters to contribute to clinical assessments.

First, the actual distance traveled by the hand from the bowl to the mouth was shorter with chopsticks than with a spoon. Furthermore, the hand holding the chopsticks moved at a slower speed. This slowness might have been caused by the shorter transfer distance [29] observed in the chopstick condition than in the spoon condition. These shorter and slower hand transfers might be due to the larger forearm rotations and smaller shoulder flexion and abduction motions associated with chopstick use, as noted earlier. In contrast, when transporting food with a spoon, the larger hand transfer distance and higher speed might be due to increased shoulder motions rather than forearm movements. Thereby, the effective hand spatiotemporal transfers during the transporting phase might be mainly caused by shoulder joint motion and not forearm joint motion. Contrastingly, forearm joint motion might affect well transfer of the utensil tip rather than the hand.

The relative distance (ie, the ratio between the actual and theoretical shortest distance) for the hand with chopsticks was greater than for the hand with the spoon, indicating lower efficiency in the hand spatial transfer. This also suggests that forearm rotation might make the movement in the tip of chopsticks efficient, not in the hand holding chopsticks. However, the relationship between hand spatiotemporal parameters as well as utensil tip motion and joint angles is beyond the scope of this study and considered an area for future research.

Additionally, we analyzed the timing of maximum velocity (motor control strategy) and number of movement units (smoothness) and found no obvious differences between utensil conditions. The comparable reaching strategies and movement smoothness between conditions might indicate that the healthy participants in this study had well-learned motor control for these modes of feeding. These characteristics are generally assessed in patients [13]; this study provides valuable data on comparable motor control and smoothness between 2 utensil-food type conditions in normal hand motions regarding such assessments.

### Clinical Implications

Clinicians might determine appropriate eating utensils used by patients based on their ability to use them, considering factors such as paresis and intellectual ability [2]. Rheumatoid arthritis [30] and Parkinson disease [31] may also be included in target populations as they have altered upper body kinematics during functional tasks. The assessment can refer to the findings of this study regarding joint angle positions, ranges of joint motion, and hand spatiotemporal parameters as fundamental normal movement knowledge, which could support clinical judgments when selecting chopsticks for eating solid foods or a spoon for eating semisolid foods in East Asian countries. For instance, individuals with limited shoulder flexion or abduction or increased elbow flexion angles might be suited to using chopsticks to eat solid foods, which include small-sized pickles used in this study, bite-sized meats and vegetables, or sticky rice; conversely, those who demonstrate significant forearm rotation during the table phase might benefit from using a spoon to eat semisolid foods, which include yogurt, jelly dessert, or rice porridge. Movements for using a spoon to eat semisolid foods may also be consistent with those for using a spoon to eat liquid foods such as soup or solid foods such as separated rice and finely chopped vegetables. These combinations of utensils and foods, alongside the typical movements currently revealed, would be realistic about food customs in East Asia. Additionally, differences in hip flexion positions and motions (ie, trunk inclination) may not be related to utensil selection.

In the rehabilitation of eating difficulties in East Asian countries, when changing food forms from semisolid to solid, patients’ forearm supination should be well confirmed for using chopsticks. Compared with the spoon condition, chopstick use typically involves shorter and slower hand movements during the transporting phase, which can be considered normal. If the nondominant hand is trained for chopstick use with solid foods, improvements as spoon use with semisolid foods in control strategy and movement smoothness—similar to those observed in this study—could be assessed, as recently reported [32,33]. Hand spatiotemporal parameters involving nondominant or impaired hands using utensils could also be evaluated based on the current findings. Although male participants showed greater neck right rotation positions and motions and female participants showed smaller wrist dorsiflexion positions in chopstick condition than in spoon condition should be assessed as individuals with normal state in each disease, these sex-related characteristics would be emphasized particularly in diseases with ununiform sex ratio such as collagen disease and cervical spondylotic myelopathy.

### Limitations and Future Research

The present results were derived from measurements obtained during specific movement tasks using an inertial motion-capturing system, which may limit the generalizability of the findings to feeding movements observed in clinical settings. Future studies should investigate whether certain foods could be more prone to being dropped by examining the effects of different eating utensils (eg, chopsticks with supportive features, forks, and spoons) and various types of foods (eg, viscosity, size, and forms) separately, as these may be confounding factors in the current experiments; moreover, these studies should define these effects on feeding movements. This approach might help verify and expand upon the current findings. Drawing connections between joint motions and the hand spatiotemporal parameters and utensil tip motions, despite being out of this study objectives but discussed, might also be a valuable method for more deeply understanding the differences between the current 2 utensil-food type conditions; thus, eating movements would be assessed well. Moreover, future research should also involve populations with upper body dysfunctions.

### Conclusions

This study investigated upper body joint angles and hand spatiotemporal parameters during feeding movements involving chopsticks for solid food and a spoon for semisolid food across 4 feeding phases and both sexes. The results indicate that chopstick use, regardless of sex, generally requires smaller shoulder and neck joint angle positions and motions across phases but involves larger elbow and forearm joint motions based on the phase. Additionally, when transporting food from the bowl to the mouth using chopsticks, the hand travels a shorter distance and moves slowly. Spoon use, in contrast, involves larger elbow, forearm, and wrist ulnar deviation motions when taking up semisolid food from the bowl. Regarding sex differences, male participants exhibit increased right neck rotation positions and motions when using chopsticks compared with using a spoon, whereas female individuals demonstrate reduced wrist dorsiflexion positions under the chopstick condition relative to the spoon condition. These movement characteristics, influenced by the combination of utensils and food types specific to East Asia countries, could facilitate clinical assessments of feeding behaviors specific to food customs in individuals with upper body dysfunction and diseases specific to each sex and aid in determining adaptive utensils.
